# Rapid generation of human recombinant monoclonal antibodies from antibody-secreting cells using ferrofluid-based technology

**DOI:** 10.3389/fimmu.2024.1341389

**Published:** 2024-04-18

**Authors:** Veronica Strazza, Marco Rossi, Andrea Avati, Giusy Tiseo, Marco Falcone, Maria Grazia Cusi, Francesco Menichetti, Paola Ricciardi-Castagnoli, Cristina Tinti, Piero Pileri

**Affiliations:** ^1^ Hyper Antibody Research & Development (HARD) -Lab, Toscana Life Sciences Foundation, Siena, Italy; ^2^ Department of Biotechnology, Chemistry and Pharmacy, University of Siena, Siena, Italy; ^3^ Infectious Diseases Unit, Department of Clinical and Experimental Medicine, Azienda Ospedaliero Universitaria Pisana, University of Pisa, Pisa, Italy; ^4^ Department of Medical Biotechnologies, University of Siena, Siena, Italy

**Keywords:** human monoclonal antibodies, antibody secreting cells (ASCs), single cell PCR, CD138-ferrofluid, transcriptionally active PCR (TAP), antigen-specific ASCs, functional selection, COVID-19

## Abstract

Monoclonal antibodies (mAbs) are one of the most important classes of biologics with high therapeutic and diagnostic value, but traditional methods for mAbs generation, such as hybridoma screening and phage display, have limitations, including low efficiency and loss of natural chain pairing. To overcome these challenges, novel single B cell antibody technologies have emerged, but they also have limitations such as *in vitro* differentiation of memory B cells and expensive cell sorters. In this study, we present a rapid and efficient workflow for obtaining human recombinant monoclonal antibodies directly from single antigen-specific antibody secreting cells (ASCs) in the peripheral blood of convalescent COVID-19 patients using ferrofluid technology. This process allows the identification and expression of recombinant antigen-specific mAbs in less than 10 days, using RT-PCR to generate linear Ig heavy and light chain gene expression cassettes, called “minigenes”, for rapid expression of recombinant antibodies without cloning procedures. This approach has several advantages. First, it saves time and resources by eliminating the need for *in vitro* differentiation. It also allows individual antigen-specific ASCs to be screened for effector function prior to recombinant antibody cloning, enabling the selection of mAbs with desired characteristics and functional activity. In addition, the method allows comprehensive analysis of variable region repertoires in combination with functional assays to evaluate the specificity and function of the generated antigen-specific antibodies. Our approach, which rapidly generates recombinant monoclonal antibodies from single antigen-specific ASCs, could help to identify functional antibodies and deepen our understanding of antibody dynamics in the immune response through combined antibody repertoire sequence analysis and functional reactivity testing.

## 1 Introduction

Monoclonal antibodies are an important class of therapeutic molecules for the treatment of serious human diseases, with therapeutic indications spanning oncology, immunology and infectious diseases. More than 100 monoclonal antibodies have already been approved for use in the U.S. or Europe (1), and their number is growing exponentially. Human mAbs can be produced by a variety of methods, including immortalization of B cells with Epstein-Barr virus and the production of B cell hybridomas (2, 3), humanization of antibodies from other species (4), use of phage display libraries (5), or recombinant production of antibodies from isolated single B cells (6, 7). Display methods have been widely adopted as a technology for the production of monoclonal antibodies. A significant limitation of most display systems is that libraries are constructed by randomly combining antibody variable region genes, which typically results in the loss of natural cognate pairings that are evolved and selected for *in vivo* during an immune response. Consequently, unnatural variable region pairs combine unproductively, resulting in reduced specific diversity. Fully human antibodies can also be generated using hybridoma technology in transgenic mouse models, such as the HuMab mouse and the XenoMouse, in which the mouse immunoglobulin (Ig) gene loci have been replaced with human loci within the transgenic mouse genome (8). However, since the number of B cells in a mouse at any given time is ≈10^8^, and the theoretical diversity of the human antibody repertoire exceeds 10^11^, an individual mouse can harbor only a fraction of the potential human repertoire (9, 10). The culture of single antigen-specific memory B cells has also been successfully used as a method for the production of monoclonal antibodies. However, it has drawbacks because it requires 10-15 days of *in vitro* differentiation of isolated single antigen-specific memory B cells into antibody-secreting cells (ASCs) and the use of an expensive cell sorter.

Crucially, the antibodies present in serum are not produced directly by memory B cells, but by ASCs. Since plasma cells are responsible for the production of the vast majority of circulating immunoglobulins, this terminally differentiated B cell subset represents an excellent source of high-quality antibodies. Although it is an attractive possibility to derive mAbs from ASCs of selected individuals with high antibody titres, a major challenge is that, unlike memory B cells, ASCs cannot be selected for antigen binding due to low (IgA, IgM, IgE) or absent (IgG) surface expression of B cell receptors (BCRs) (11), and it is necessary to implement an alternative selection method to identify rare antigen-specific ASCs.

Ferrofluid is a colloidal fluid containing nanoscale ferromagnetic particles dispersed in a carrier fluid. These particles, typically made of materials such as iron oxide, are coated with a surfactant to prevent clumping. Due to the presence of these nanoparticles, ferrofluids exhibit exceptional superparamagnetic properties, exhibiting strong magnetic properties when exposed to an external magnetic field. Ferrofluid nanoparticles are much smaller than microbeads, resulting in a greater surface area to volume ratio. This increased surface area facilitates superior antibody or ligand binding, thereby increasing their ability to capture target cells from a given sample. In 2011, the CellSearch System was patented as a method to capture and detect rare circulating plasma cells (CPC) and abnormal plasma cells or multiple myeloma cells (CMMC) using an anti-CD138 FerroFluid conjugated antibody in order to detect, monitor and characterize CMMC diseases, including monoclonal gammopathy of undetermined significance (MGUS) and Smoldering multiple myeloma (SMM) (12, 13). In addition, we sought to use the Ferrofluid technology in a completely different application to recover single and rare antigen-specific plasma cells from human peripheral blood samples. Plasma cells are found in very low numbers in the peripheral blood of healthy donors (14), and only a small fraction of them is specific for a particular antigen. Our approach for the identification of antigen-specific ASCs is based on two steps: first, the recovery of the entire population of CD138-positive ASCs from human peripheral blood mononuclear cells (PBMCs) by magnetic sorting, followed by live screening of cell culture supernatants for antigen-specific Ig-secreting cells.

Here, we propose to combine live antigen-specific screening of CD138-Ferrofluid-enriched ASCs with a high-throughput “mini-gene” approach for rapid expression and analysis of antibodies from the blood of antigen-experienced individuals, and have recruited COVID-19-recovering patients to demonstrate feasibility.

## 2 Materials and methods

### 2.1 Recruitment of SARS-CoV-2 convalescent donors and specimen collection

The Azienda Ospedaliero Universitaria Pisana - UO Malattie Infettive provided samples from SARS-CoV-2 convalescent donors of both sexes who gave written consent. The study was approved by the local ethics committee (Ethics Committee CE AVNO - Regione Toscana; Authorization #17761) and was conducted in accordance with good clinical practice as defined by the Declaration of Helsinki (European Council 2001, US Code of Federal Regulations, ICH 1997). Blood samples (≈40 mL) were collected from convalescent adult blood donors 15-44 days after the onset of SARS-CoV-2 infection. Convalescent patients were considered eligible for the study if two consecutive nasopharyngeal swabs were negative for SARS-CoV-2. PBMCs were then purified from human blood using Ficoll-Paque PLUS density gradient centrifugation medium. Whole blood was diluted with an equal volume of Dulbecco’s Phosphate Buffered Saline (DPBS) (Gibco), layered on a volume of Ficoll-Paque PLUS (Cytiva), then centrifuged at 600×g for 30 min at room temperature with acceleration and deceleration set to zero. PBMCs were collected from the Ficoll-Paque Plus-plasma interface in a 15 mL tube and washed with DPBS (800×g, 10 min, 4°C).

### 2.2 Enrichment of ASCs from PBMCs

Enriched plasma cell preparations were obtained from PBMCs using the reagents provided with the CellSearch™ CMMC enumeration system (Menarini Silicon Biosystems) with slight modifications. In detail, 40x10^6^ PBMCs were diluted in 2 mL DPBS plus dilution buffer with 150 µL capture enhancement reagent and anti-human CD138 conjugated to ferrofluid particles. Enrichment was achieved by three steps of incubation for 10 minutes, plus 10 minutes and another 20 minutes in a quadrupole magnetic separation system (QMS17, Immunicon Corp). Each step was interspersed with gentle agitation of the cells and reagents present in the tube. At the end of the incubations, CD138-positive cells adhered to the tube wall, while unbound cells were removed with a Pasteur pipette without touching the tube wall in contact with the magnet. A wash step was then performed by adding 3 mL of wash buffer (DPBS plus binding buffer) to the tube, followed by incubation in the magnetic separation system for 10 minutes. The tube was then removed from the magnet to collect the positively selected cells, which were resuspended in 224 µL of a mixture of equal volumes of DPBS and dilution buffer. Cell-bound ferrofluid particles were removed by a final incubation with biotin-containing buffer (included in the Menarini Silicon Biosystems kit) for 20 minutes at room temperature in the dark, then it was washed with DPBS to finally obtain a cell population highly enriched for CD138^+^ cells.

### 2.3 Identification of antigen-specific ASCs

The CD138^+^ enriched cells were counted and plated at 50 cells per well in low volume 384-well tissue culture treated microplates (Corning) in RPMI-1640 medium (Sigma-Aldrich) supplemented with 10 ng/mL human IL-6 (Sigma-Aldrich) and the supernatant of M2-10B4 feeder cells (ATCC CRL-1972). The next day, the supernatants of these cells were tested for antigen specificity using a spike-specific enzyme-linked immunosorbent assay (ELISA). The wells that were positive for our antigen of interest were replated in the same condition by limiting dilution to have one cell per well. On the third day, the cells secreting spike-specific immunoglobulins were identified by the specific ELISA assay and then these cells were harvested and preserved in a specific Lysis Buffer (UltraPure™ DNAse/RNAse-Free Distilled H_2_O (Invitrogen), 10X sterile PBS, 0.1M DTT (Promega) and 40 U/μL RNasin™ Plus RNase Inhibitor (Promega).

### 2.4 SARS-CoV-2 spike protein cloning, expression and purification

The spike (S) glyco-protein of SARS-CoV-2 is a trimeric class I fusion protein that exists in a metastable prefusion conformation that undergoes a substantial structural rearrangement to fuse the viral membrane with the host cell membrane. A human codon-optimized nucleotide sequence coding for a soluble version of the S protein (amino acids 1–1208; GenBank: MN994467) including the T4 foldon trimerization domain, a histidine-tag and a strep-tag, was commercially synthesized (GeneArt) and cloned into the mammalian expression vector pcDNA 3.4. The protein sequence was modified to remove the polybasic cleavage site (RRAR to GSAS), and two stabilizing mutations were also introduced (K986P and V987P).

The recombinant protein was produced by transient transfection of ExpiCHO-S cells (Gibco) using ExpiCHO Expression System Kit (Gibco) in 250mL non-baffled flasks (Corning), according to the manufacturer’s protocol. Supernatant from transfected cells was harvested on day 8 post-transfection and the recombinant protein was purified using His Trap HP Ni Sepharose High-Performance nickel-charged IMAC resin (Cytiva). The appropriate fractions containing recombinant protein were dialyzed against phosphate buffered saline (PBS) pH 7.4 using Slide-A-Lyzer MINI Dialysis Device, 20K MWCO (Thermo Scientific) in agitation at 4°C. The final protein concentration was determined by measuring the colorimetric response at 562 nm using the Pierce BCA protein assay kit (Thermo Scientific), following the manufacturer’s instructions.

### 2.5 ELISA assay for the identification of antigen-specific individual ASCs

384-well flat-bottom microplates from PerkinElmer, which have a high binding capacity, were used. A volume of 10 µL per well of SARS-CoV-2 spike glycoprotein was applied and the plates were left to incubate overnight at 4°C. Plates were washed three times with 100 µL/well of Washing Buffer (PBS (Gibco), 0.05% Tween-20 (Sigma-Aldrich) and saturated with 35 µL/well of Blocking Buffer (PBS, 1% bovine serum albumin (Fisher Scientific), 1% fetal bovine serum (FBS) (Gibco)) for 1 hour at 37°C. After three washes, 12µL of cell culture supernatant was added to the plate. After incubation for 1 hour at 37°C, the plates were washed four times and incubated again for 1 hour at 37°C with 20 µL of goat anti-human IgG (H+L) HRP conjugate antibody (Invitrogen) diluted 1:3,500 in Blocking Buffer. The plates were washed six times and 20 µL of 1-Step™ Ultra TMB (3,3′,5,5′-tetramethylbenzidine)-ELISA Substrate Solution (Thermo Scientific) was added and incubated for 20 min at RT in the dark, followed by the addition of 20 µL of 0.5M HCl. Absorbance was then measured at 450 nm using a Spectramax M2 Microplates Reader. The threshold for sample positivity was set at twice the optical density (OD) of the blank.

### 2.6 Reverse transcription-PCR amplification of VH and VL regions and transcriptionally active PCR

Single antigen-specific ASCs were isolated and lysed in 4 µL of Lysis Buffer (see Section 2.3). RNA from 4 µL of single cell lysate was reverse transcribed (RT) in a 20 µL reaction volume using Superscript IV reverse transcriptase (Invitrogen). The RT reaction was performed by a first annealing step at 72°C for 3 min, followed by incubations at 42°C for 10 min, 25°C for 10 min, 50°C for 1 h, 94°C for 5 min and left at 4°C until the next step.

A 5 µL portion of the complementary DNA (cDNA) was preamplified in a 20 µL reaction with Terra™ PCR Direct Polymerase (Takara Bio) and IS-PCR primer [AAGCAGTGGTATCAACGCAGAGT] (Eurofins Genomics), following the manufacturer’s instructions, to increase the total amount of genetic material while minimizing amplification bias (15). The preamplification process consisted of an initial denaturation at 98°C for 3 minutes, followed by 18 cycles of 98°C for 15 seconds, 65°C for 30 seconds, 68°C for 4 minutes, and a final extension at 72°C for 10 minutes.

The variable regions of the immunoglobulin heavy (IgH) and light chains (Igκ and Igλ) genes were amplified using two nested PCR reactions with primers adapted from Tiller et al. (6). Primer nucleotide sequences are listed in **Supplementary Table 1**. All PCR reactions were performed in 25 µL volumes containing 200 nM of each primer or mix, 1x buffer for KOD Hot Start DNA Polymerase (Merck), 2.5 mM MgSO_4_, 300 µM of each dNTPs (Invitrogen), 0.5 U KOD DNA Polymerase (Merck), and 0.1 U Taq DNA Polymerase (Invitrogen). 3 µL of cDNA or pre-amplified cDNA were added in the first PCR. The second round of PCR was carried out using 3 µL of the unpurified first round PCR product. The amplification protocols were standardized as follows: the heavy and κ light chains underwent 50 cycles, while the λ light chains underwent 40 cycles, following an initial activation step at 95°C for 3 minutes. Each cycle included a 30-second denaturation step at 95°C, a 30-second annealing step at 58°C or 60°C for heavy and κ light chains, and λ light chains respectively, and a 1-minute extension step at 72°C. The process concluded with a final extension step at 72°C for 10 minutes.

**Figure 1:**
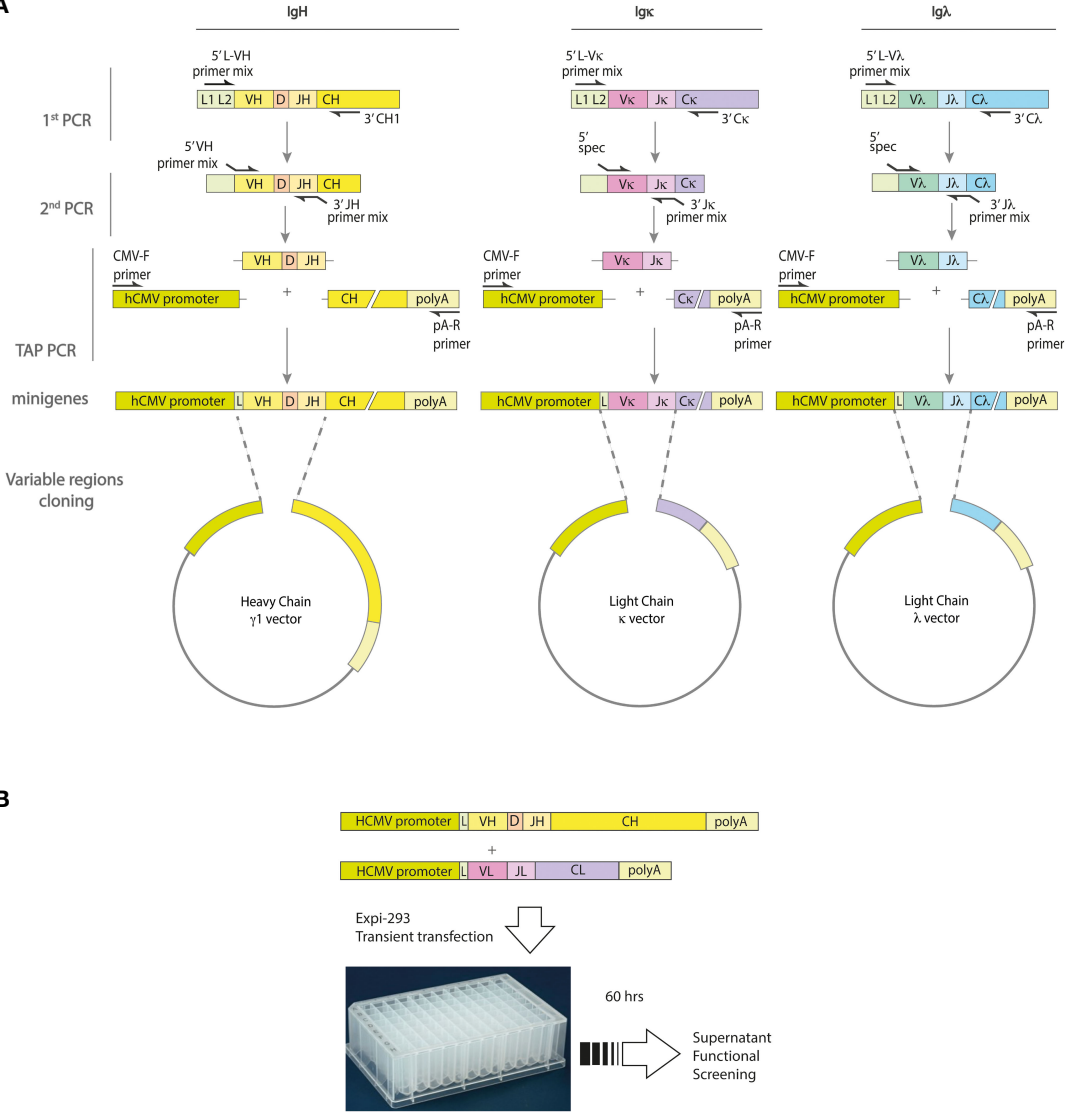
Strategy for minigenes assembly and expression of human monoclonal antibodies. **(A)** The variable region genes of the IgH and IgL chains are obtained from single cells by a two-step nested RT-PCR procedure involving random hexamer retro-transcription and subsequent amplification. The first round of PCR amplification uses a mixture of forward primers specific for the leader region and reverse primers specific for the respective IgH, Igκ or Igλ constant region. A second round of PCR is then performed using primer mixes specific for the V (variable) and J (joining) genes. These primers also contain adaptor sequences that allow the minigenes to be assembled by a third PCR. The resulting minigenes contain the hCMV promoter and constant regions, as well as the polyadenylation site. **(B)** To validate the re-production of antigen-specific monoclonal antibodies, Expi-HEK293 cells are transfected with the IgH and IgL minigenes, and the cell culture supernatants are tested in functional assays.

PCR was then used to produce transcriptionally active (TAP) linear DNA fragments (minigenes) for both the heavy and light chains. These fragments consist of the Ig variable region (VH or VL), a constant region fragment containing a poly-A signal sequence, and the human cytomegalovirus (hCMV) promoter region. They are useful for direct transfection into mammalian cells to produce recombinant immunoglobulins for validation screening. To achieve this goal, the plasmids AbVec2.0-IGHG1 (Addgene;#80795), AbVec1.1-IGKC (Addgene;#80796), and AbVec1.1-IGLC2-XhoI (Addgene;#99575) (16) were firstly linearized by PCR using the primers listed in **Supplementary Table 2**. To prepare the linearized plasmids, 10 ng of each plasmid template underwent 35 PCR cycles after an initial activation step at 95°C for 3 minutes. Each cycle consisted of a 30-second denaturation step at 95°C, followed by a 30-second annealing step at 55°C, and a 5-minute extension step at 72°C. The process was concluded with a final extension step at 72°C for 10 minutes. The TAP minigenes were prepared by using 10 ng of linearized plasmids and 2 µl of PCR product of the Ig variable regions in a PCR reaction with the CMV-F and polyA-R primers (**Supplementary Table 2**), as shown in **Figure 1**. The amplification was performed by an initial activation step at 95°C for 3 minutes, followed by 35 cycles of 95°C for 30 seconds, 55°C for 30 seconds and an extension step at 72°C for 1 minute. The process was completed with a final extension step at 72°C for 10 minutes.

### 2.7 Recombinant antibody production by transient expression of TAP in the Expi 293 system

Using the ExpiFectamine™ 293 Transfection Kit (Gibco), Expi-HEK293F™ cells (Gibco) were transiently transfected with either paired transcriptionally active heavy and light chain PCR fragments or plasmid DNA encoding cloned antibodies at a 1:2 ratio. The cells were cultured for one week at 37°C with shaking at 125 rpm and 8% CO_2_. ExpiFectamine™ 293 Transfection Enhancers 1 and 2 were added 18-22 hours post-transfection based on the manufacturer’s protocol to enhance both transfection and protein expression. The supernatants from the cell culture were collected at day 3 and day 6 post-transfection. Next, the cells were centrifuged at 400 g for 5 minutes. The collected supernatants were further analyzed.

### 2.8 ELISA quantification of human IgGs

The secretion of human IgGs by individual ASCs or recombinant monoclonal antibodies present in transiently transfected Expi-HEK293 cell supernatant was quantified using an ELISA assay. Recombinant Cetuximab (Merck) was used as the standard. Spectra Plate 384 High-Binding Plates were coated with 10 µL/well of unconjugated goat anti-human IgG Fc (Invitrogen) at a concentration of 1 µg/mL. The plates were incubated at 4°C overnight. After incubation, the plates were washed three times with Wash Buffer. Thirty-five microliters of Blocking Buffer was added to each well. The plates were incubated at 37°C for 1 hour. Following three washes, serially-diluted supernatants or the serially-diluted standard were added. The blank was prepared with the blocking buffer. After incubating for 1 hour at 37°C, the plates were washed four times. Then, 20 µL of goat anti human IgG H+L HRP-conjugated antibody (Invitrogen) diluted 1:5,000 in Blocking Buffer were added to each well. The plates were then incubated for an additional hour at 37°C and washed six more times. Then, 20 µL of the 1-Step™ Ultra TMB ELISA Substrate Solution were added and incubated for 15-20 minutes in the dark at room temperature. Finally, 20 µL of 0.5 M HCl were added. The level of absorbance was quantified at a wavelength of 450 nm. The concentration of immunoglobulins in the supernatant was determined by extrapolating the sample values using the standard curve.

### 2.9 Assessment of SARS-CoV-2 virus neutralization

The neutralization assay for SARS-CoV-2 virus was conducted on Vero E6 cells (ATCC CRL-1586) in a 96-well microplate. Two-fold serial dilutions (1:4 to 1:1024) of mAbs, each at 25 microliters, were combined with an equivalent volume of SARS-CoV-2 WT strain (SARS-CoV-2/human/ITA/Siena-1/2020; GenBank: MT531537.2), Delta (B.1617.2) (SARS-CoV-2/human/ITA/TUS-Siena-40/2021; GenBank: OM736177.1), or Omicron (BA.1) (SARS-CoV-2/human/ITA/TUS-Siena5324294/2022; GenBank: OM956353.1), each containing 100 TCID_50_. This was followed by incubating the mixture at 37°C for 90 minutes. A final addition of 50 μL of Vero E6 cell suspension (2x10^5^ cells/mL) prepared in complete DMEM (Lonza) was made to each well. The cultures were incubated at 37°C and examined daily under an Olympus IX51 microscope to identify the presence of cytopathic effects (CPE). The Reed-Muench method (17) was used to calculate the 50% endpoint titer, and the assay included positive and negative control serum. The geometric mean titers (GMTs) for the neutralization assays were calculated.

### 2.10 Monoclonal antibody repertoire analyses

VH and VL sequence reads of monoclonal antibodies were manually curated and retrieved using CLC Main Workbench (Qiagen). The analyzed reads were saved in FASTA format, and the repertoire analyses were performed using standalone IgBlast (18). Comparison analysis was performed in Python using NumPy (https://numpy.org/) and Pandas (https://pandas.pydata.org/), while figures were produced using the Matplotlib tool (https://matplotlib.org/) and Seaborn (https://seaborn.pydata.org/).

## 3 Results

### 3.1 Development of a Ferrofluid-based method for the isolation of ASCs

**Figure 2:**
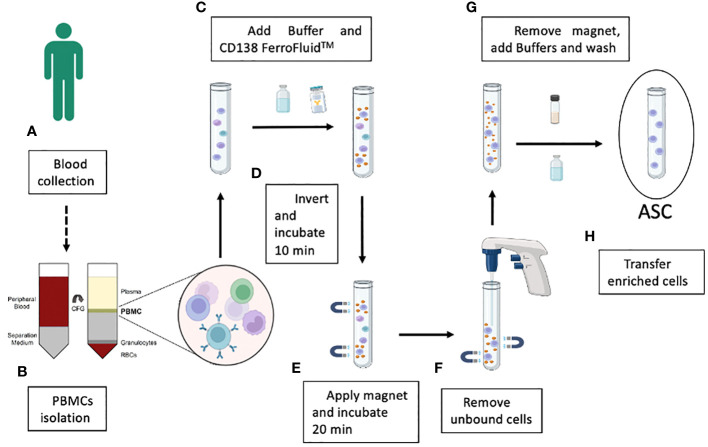
Schematic illustration of the key steps in the enrichment of the blood ASC population by using CD138-coupled magnetic nanoparticle technology. Blood samples are collected from convalescent donors **(A)** and PBMCs are separated from red blood cells, granulocytes and other plasma components by a Ficoll™ gradient **(B)**. After the addition of a specific FerroFluid buffer, PBS and the CD138-FerroFluid capture reagent for the enrichment procedure **(C)** and several steps of mixing and incubation in the magnet **(D, E)**, the non-enriched cells are removed **(F)**. The CD138-positive selected ASCs are incubated with a specific buffer to remove the FerroFluid nanoparticles **(G)**, and the enriched ASCs are collected **(H)**.

The enrichment of CD138^+^ cells by Ferrofluid (CD138-FF) technology was developed as a diagnostic tool (CellSearch) to isolate circulating tumor cells in multiple myeloma patients using 4-6 mL of fresh blood (13). Instead, we wanted to use the CD138-FF technology in a completely different application to isolate single and rare antigen-specific ASCs from human peripheral blood samples and to develop recombinant antibodies from them. ASCs can be identified by their very high expression of CD27 and CD38 surface markers as well as CD138 (syndecan-1) (19). Since the frequency of CD138^+^ cells among PBMCs in normal donors is estimated to be 1 x 10^−4^ (20), it may be difficult to obtain a useful number of antigen-specific recombinant monoclonal antibodies using the original protocol of 4-6 mL of whole blood. To overcome this problem, we adapted the original protocol to the use of purified PBMCs as starting material, thereby improving the yield of recovered ASCs. The strategy used to obtain an enriched population of ASCs from blood is shown in **Figure 2**. Typically, 5x10^3^ CD138-enriched cells could be obtained from 1 mL of blood using this method. To understand whether the recovered CD138-enriched cells could be used to screen for antigen-specific monoclonal antibodies, their ability to secrete antibodies was tested in a functional assay *in vitro* by establishing single cell cultures and testing the supernatants in a quantitative ELISA. After 16 hours of culture, 4% of the culture supernatants contained detectable amounts of human IgGs above the 200 ng/mL sensitivity of our assay, as shown in **Supplementary Figure 1**, demonstrating the feasibility of our approach.

### 3.2 Identification of individual ASCs that specifically recognize the SARS-CoV-2 spike glycoprotein

**Figure 3:**
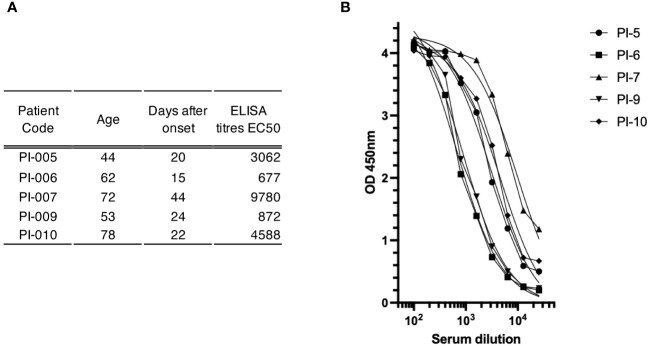
Antibody-mediated immune response to spike glyco-protein in study subjects. **(A)** Serum sample characterization. This section shows a compilation of serum samples along with their respective attributes, including patient identifiers, age, duration in days from disease onset, and ELISA titers expressed as the half-maximal effective concentration (EC_50_). **(B)** Plot of the ELISA titration curve of different patient sera (legend at top right) against the trimeric spike glyco-protein; the serum dilution is indicated on the x-axis, while the OD value is indicated on the y-axis.

To gain insight into the suitability of our method for identifying antigen-specific ASCs, we used the blood of convalescent patients recovering from COVID-19. Five subjects aged 44-78 years were enrolled in this study between February and April 2021 after negative nasopharyngeal swab. Blood samples were collected 15-44 days after the onset of SARS-CoV-2 infection and their sera were tested for the presence of spike-specific antibodies using an ELISA assay against a soluble trimeric stabilized spike glycoprotein (21). As shown in **Figure 3**, all convalescent donors had antibodies that recognized the recombinant spike glycoprotein in an ELISA assay. Titers (EC50) ranged from 1:676 to 1:9780. ASCs from the five donors were enriched with CD138 antibody conjugated to FerroFluid from the blood-purified PBMCs, and the average yield of the ASC-enriched preparations was 5.7x10^3^ cells (range 1.3-10 x10^3^) per 1 mL of source blood, as shown in **Table 1**. The CD138 enriched cells were then subjected to a two-step screen to select single spike-specific ASCs. First, the CD138-FF enriched preparations were plated at 50 cells per well, allowed to secrete antibodies for 16 hours, and then the supernatants were tested by ELISA for spike glycoprotein recognition. After the initial screening, each ELISA-positive pool was replated at a limiting dilution to reach the single cell level and reassayed for antigen specificity. Using this procedure, we screened 4.4x10^5^ CD138-FF enriched cells from the five convalescent subjects and identified a total of 132 individual ASCs (**Table 1**) that specifically recognized the spike glycoprotein. On average, there were 3 spike-specific plasma cells per 10^4^ cells in the ASC-enriched preparation.

**Table 1:** Yields of ASC-enriched PBMC-derived preparations.

Source ID	Days from disease onset	Blood obtained (mL)	Isolated cells			
			Recovered PBMCs (x10^6^)	Recovered CD138^+^ ASCs (x10^3^)	ASCs/µL blood	‰ ASCs in PBMCs
PI-005	20	44	117	272	6,2	2,3
PI-006	15	40	42	167	4,2	4,0
PI-007	44	40	62	280	7,0	4,5
PI-009	24	40	90	50	1,3	0,6
PI-010	22	40	68	400	10,0	5,9

### 3.3 Amplification of immunoglobulin variable region transcripts from single ASCs, assembly of minigenes for expression

For the rapid production of recombinant monoclonal antibodies from single antibody-secreting cells, we have implemented a strategy as shown in **Figure 1A**. The approach involves amplification and cloning of full-length IgH and IgL (Igκ or Igλ) variable regions into transcriptionally active minigenes containing the human Igγ1, Igκ, or Igλ constant regions.

To provide an unbiased approach to the analysis of the expressed genes of the IgH and IgL chains, we generated cDNA from single B cells using oligo-dT and random primers. Furthermore, a pre-amplification step was included to enhance the possibility of obtaining coding sequences for variable regions of immunoglobulin from an individual cell. Immunoglobulin heavy (IgG) and light (κ and λ) chains were then amplified by two round-nested PCRs using primers adapted from Tiller et al. (6) (see **Supplementary Table 1**). The results obtained show that both the κ and λ light variable regions, in addition to the heavy chains, were effectively rescued by this process (**Supplementary Figure 2**).

To assess the suitability of this method for frozen samples, we processed antigen-specific ASCs from fresh or frozen PBMCs collected from the same donor. The findings indicated that variable regions of the immunoglobulin can be retrieved with a similar frequency from frozen and fresh samples, which supports the application of frozen samples with similar yields (see **Supplementary Figure 3**). Although the pre-amplification step enhanced the quality and quantity of amplified product, it did not result in a substantial increase in the number of amplified variable regions. We used transcriptionally active PCR linear DNA fragments, known as “minigenes,” to express both the heavy and light chains, thereby streamlining the process and avoiding labor-intensive cloning techniques. To construct these minigenes, we used a tertiary PCR to combine an hCMV promoter, the Ig variable region DNA fragment, and a constant region fragment containing a polyadenylation sequence (see **Figure 1A**). As a result of this process, we generated two separate minigenes: one encoding the heavy chain and the other encoding the light chain (see **Supplementary Figure 4**).

Because of the exploratory design of this study, a limited number of single antigen-specific ASCs were utilized in constructing the heavy and light chain minigenes. We obtained paired heavy and light chains from 36 out of 58 single antigen-specific ASCs where Ig variable region rescue was attempted, indicating approximately 60% yield of individual ASCs (refer to **Table 2**).

**Table 2:** Paired minigene yields from single antigen-specific ASCs.

Source ID	ASC input (x10^3^)	Single Antigen-Positive ASC	Used for VH and VL Amplification (*)	Paired mini-genes recovery
PI-005	50	6	6	3
PI-006	105	17	17	11
PI-007	20	1	1	0
PI-009	25	28	8	7
PI-010	240	80	26	15
Total	440	132	58	36 (**)

(*) Due to the exploratory nature of the study, only a subset of the enriched ASCs was used to isolate single antigen-specific ASCs and a subset of single antigen-specific ASCs was used to assemble the heavy and light chain minigenes. (**) Paired heavy and light chains were obtained from approximately 62% of the antigen-specific ASCs.

### 3.4 Validation and characterization of the produced monoclonal antibodies.

**Figure 4:**
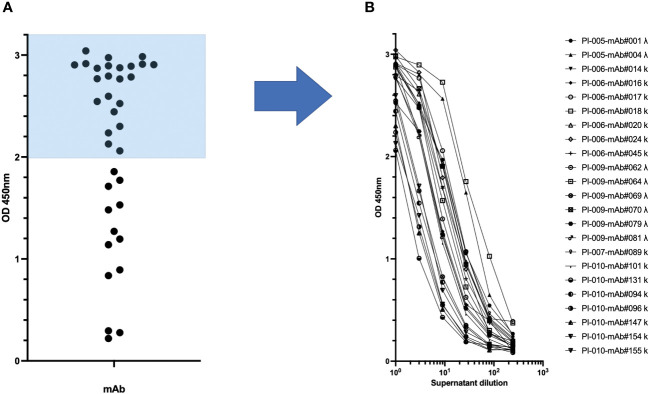
Production of recombinant spike-specific immunoglobulins by transient transfection of TAP minigenes. **(A)** The specificity of the supernatants obtained from Expi-HEK-293 transient transfection of TAP minigenes towards the spike glycoprotein of SARS-CoV-2 was confirmed through enzyme-linked immunosorbent assay (ELISA) in the left panel. **(B)** The most effective monoclonal antibodies were tested after dilution to demonstrate the sensitivity of their binding to dose as shown in the right panel.

The transcriptionally active minigenes encoding the heavy and light chains of each mAb were directly used for transient transfection of Expi-HEK-293 cells as shown in **Figure 1B**. The culture supernatants were then collected and tested in ELISA assays to quantify immunoglobulins and to determine their antigen-specific reactivity. Each transfection produced approximately 5 µg/mL immunoglobulin, ranging from 0.1-100 µg/mL, as shown in **Supplementary Figure 3**. The antigen-specific reactivity of the 36 recombinant monoclonal antibodies secreted in the supernatant of transiently transfected Expi-HEK-293 cells is shown in **Figure 4**. The supernatants were tested as a single point concentration (**Figure 4A**), most of the supernatants were specific, showed a dose response and retained maximum antigen recognition after a logarithmic dilution (**Figure 4B**). The minigenes can be easily converted into stable plasmids either by traditional cloning using restriction enzymes or by high-throughput ligation-independent cloning (LIC) methods (22).

### 3.5 Neutralizing activity and genetic characterization of selected monoclonal antibodies

**Figure 5:**
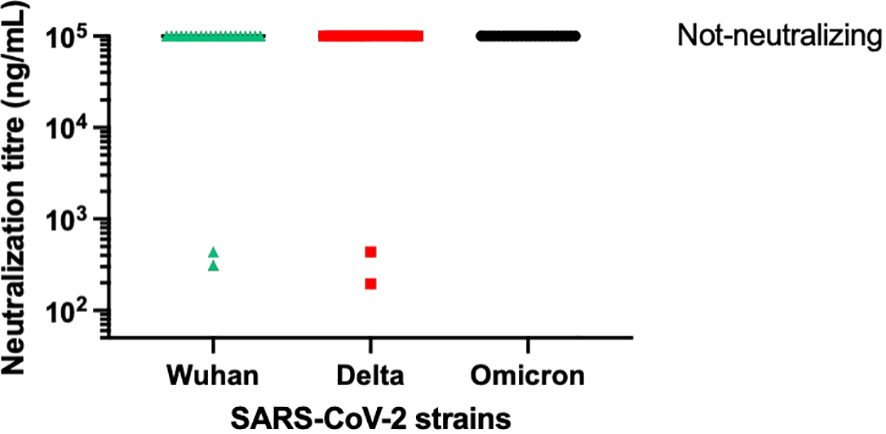
Neutralization activity of selected mAbs. The graph illustrates the neutralizing activity of 22 selected monoclonal antibodies against the original SARS-CoV-2 virus from Wuhan, as well as its Delta and Omicron variants. The supernatant from transiently transfected Expi-HEK293 cells was serially diluted, starting at a concentration of 10 µg/mL of immunoglobulins. All the monoclonal antibodies mentioned were tested for virus neutralization activity on Vero cells.

**Figure 6:**
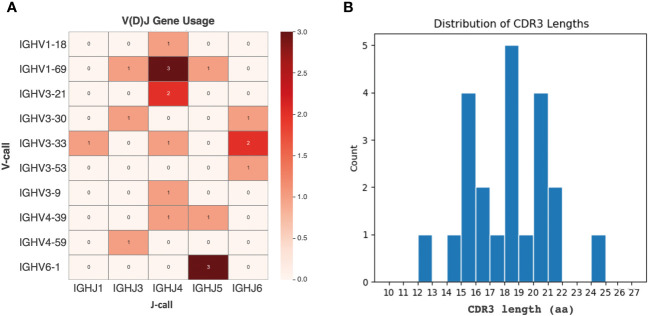
Analysis of the VH region in selected monoclonal antibodies. **(A)** IGHV and IGHJ germ line distribution. The heat map plot visually depicts the distribution of IGHV and IGHJ germline segments among the retrieved monoclonal antibodies. **(B)** CDR3 sequence length variation. The graph shows the number of complementary determining region 3 (CDR3) sequences with different lengths of amino acids (aa) among the selected mAbs. The y-axis represents the count, and the x-axis indicates the sequence length.

From the panel of 36 spike-specific mAbs, the best-performing ELISA monoclonal antibodies were tested for neutralizing activity against three strains of live SARS-CoV-2 (Wuhan, Delta, Omicron) variants. Recombinant monoclonal antibodies derived from transiently transfected Expi-HEK-293 cells were diluted to 10 µg/mL and tested for their ability to protect the layer of Vero E6 cells from the cytopathic effect induced by SARS-CoV-2 infection. Out of 22 monoclonal antibodies evaluated in this study, two (9%) were able to neutralize the authentic Wuhan and Delta viruses and prevent infection of Vero E6 cells, but none were able to neutralize the Omicron BA.1 variant, which emerged 6-9 months after the monoclonal antibodies were isolated, as shown in **Figure 5**. The variable regions of the isolated mAbs were also sequenced and analyzed for the VH gene repertoire and the length of coding region 3 (H-CDR3). **Figure 6** shows that the most frequently used VHs were IGHV1-69, IGHV3-33 and IGHV6-1, while the preferred JH was JH4. The length of H-CDR3 ranged from 12 to 24 amino acids (aa), with the majority (86%) of antibodies having a length of 15 to 20 aa, which is slightly longer than previously observed.

## 4 Discussion

During an immune response, antibodies released into the bloodstream are not produced directly by memory B cells. Instead, they are secreted by antibody-secreting cells (23). Therefore, analysis of the antigen-specific ASCs repertoire can provide valuable insights into the actual immune response. Several single B cell technologies have been developed that enable efficient sampling of the B cell repertoire (24). Despite these advances, most methods cannot screen antigen-specific plasma cells and other Ig-secreting B cell subsets, mainly due to the lack of surface Ig and the difficulty in culturing these cells.

The aim of this study was to demonstrate the efficacy of a novel method for the production of monoclonal antibodies from naturally occurring antigen-specific ASCs. The method used the CD138-FF enrichment technique in combination with functional screening of the ASC supernatant. The results of the first phase of the study showed that the CD138-FF enrichment method was successful, with 4% of the supernatants obtained from single cell cultures of the enriched population having detectable levels of human IgGs after 16 hours. This indicated that the method was effective and did not affect the function of the ASCs. In addition, the study identified 133 individual SARS-CoV-2 spike glycoprotein-specific ASCs by ELISA from 4.4x10^5^ CD138-FF enriched ASCs. This corresponds to approximately 3 antigen-specific ASCs per 10^4^ enriched ASCs, demonstrating the potential of this approach. To further improve the quality of monoclonal antibody production, various functional screening methods could be implemented. However, the production of antigen-specific ASCs may be influenced by several factors, such as the individual’s innate immune response, the immunogenicity of the antigen and the timing of sample collection, and further studies with more antigens and subjects would be useful to verify the robustness of this method.

The study also presented a strategy for obtaining immunoglobulin variable regions by PCR from single cells and inserting them into linear Ig heavy and light chain gene expression cassettes, allowing rapid expression of Ig VH and VL minigenes as recombinant antibodies from single ASCs without the need for time-consuming and labor-intensive procedures of cloning Ig into stable plasmids. This method provided large quantities of immunoglobulins by transient transfection, facilitating their functional analysis. Using this approach, thirty-six recombinant monoclonal antibodies were recombinantly produced from ASCs derived from five individuals who had recovered from COVID-19. Due to the exploratory nature of the study, only a small fraction of the available ASCs was used to generate monoclonal antibodies from selected antigen-specific ASCs. However, this technique can yield thousands of antigen-specific plasma cells from just a few milliliters of blood, demonstrating its potential for high-throughput screening of the adaptive immune response following vaccination or infection. In fact, it allows for high-throughput analysis and characterization of large numbers of samples, enabling the generation and verification of recombinant monoclonal antibodies within ten days of the initial blood collection.

The generation of mAbs from ASCs has several advantages over traditional mAb generation methods (25). Activated during an immune response, ASCs are actively involved in antibody production. The advantage of selecting and cloning ASCs that produce specific antibodies is that it increases the likelihood of developing monoclonal antibodies with functionality and potency. In contrast, the natural cognate pairings that evolve and are selected for *in vivo* during an immune response are often lost in many display systems when libraries are constructed by randomly combining antibody variable region genes. As a result, the combination of unnatural variable region pairs in an unproductive manner leads to reduced specific diversity (26). This can be a time-consuming and costly process that does not always result in the identification of effective and developable mAbs.

The monoclonal antibody generation method described in this study has the advantage of using ASCs to screen for effector function prior to antibody cloning. This step is critical for the development of effective treatments and vaccines against viral infections such as COVID-19, where the ability to isolate functional monoclonal antibodies capable of neutralizing the live virus is paramount (27, 28). Despite the limitations of using the ELISA assay in this proof-of-concept (POC) study to identify functional antibodies, the discovery of these antibodies highlights the potential of this approach to generate robust monoclonal antibodies with potent neutralizing activity. Notably, this potential is realized despite the non-selectivity of the ELISA for conformational epitopes.

Membrane-bound antibodies present on the surface of memory B cells are only suitable for screening mAbs against soluble antigens unless sophisticated engineering techniques are employed, making the screening of memory B cells for membrane-bound antigens technically challenging. In contrast, soluble antibodies secreted by ASCs are suitable for screening both soluble and membrane-bound antigens, and the development of antibodies against structurally-complex transmembrane proteins is highly desirable to facilitate the generation of potential diagnostic and therapeutic monoclonal antibodies against difficult-to-target antigens. The use of ASCs to generate mAbs is particularly well suited to advance the field of antibody-based applications and the approach described here is inexpensive and accessible and does not require specialized equipment, unlike alternatives such as the Beacon platform and microfluidics-based methods. Its simplicity and versatility make it an attractive technique for generating monoclonal antibodies with desired characteristics, highlighting its potential for adoption by any research laboratory.

The study of the immunoglobulin repertoire is a valuable technique for understanding the immune response to specific antigens and for the development of novel vaccines and treatments (29). The study of the antigen-specific repertoire involves the detection and analysis of individual antibodies produced by the immune system in response to a specific antigen. To achieve this, a large number of antigen-specific Igs must be identified and amplified using a procedure that avoids Ig cloning steps. The approach described here enables the generation of sufficient quantities of transiently expressed immunoglobulins to allow their functional characterization in a time-effective manner.

Overall, this method can select thousands of antigen-specific ASCs from a small amount of blood, suggesting the potential for high-throughput analysis of the ongoing adaptive immune response following vaccination or infection. This approach has the potential to improve researchers’ understanding of immune responses to specific antigens, and accelerate the development of breakthrough vaccines and treatments, particularly in time-critical situations.

## Data availability statement

The raw data supporting the conclusions of this article will be made available by the authors, without undue reservation.

## Ethics statement

The studies involving humans were approved by Ethics Committee CE AVNO - Regione Toscana. The studies were conducted in accordance with the local legislation and institutional requirements. The participants provided their written informed consent to participate in this study.

## Author contributions

VS: Investigation, Visualization, Writing – original draft, Writing – review & editing. MR: Investigation, Writing – review & editing, Methodology. AA: Investigation, Writing – review & editing. GT: Resources, Writing – review & editing. MF: Writing – review & editing, Resources. MGC: Investigation, Writing – review & editing. FM: Resources, Writing – review & editing. PR-C: Writing – review & editing, Funding acquisition, Resources. CT: Funding acquisition, Writing – review & editing, Project administration, Supervision. PP: Conceptualization, Investigation, Supervision, Visualization, Writing – original draft, Writing – review & editing.

## Supplementary material

Supplementary Figure 1 Evaluation of IgG secretion in ASC-enriched cultures. The quantitative ELISA graph shows the amount of IgGs produced by a single cell after 16 hours of culture, with each point representing a single result. The assay threshold is set at 200 ng/mL. Human IgG concentrations exceeding 200 ng/mL were detected in more than 4% of the culture supernatants.

Supplementary Figure 2 Immunoglobulin heavy and light chain variable regions amplified from individual ASC with and without prior cDNA amplification. The amplification products of the immunoglobulin heavy chain (IgH) (top row) and light chains κ and λ (middle and bottom rows, respectively), resulting from the second round of nested PCR, are shown on agarose gels. The left panel of the figure shows the PCR products derived from the cDNA of individual cells, while the right panel shows the amplification resulting from the addition of a pre-amplification step to augment the input material.

Supplementary Figure 3 Comparison of the recovery of immunoglobulin heavy and light chain variable regions from single antigen-specific ASCs in fresh and frozen PBMCs from the same individual. Agarose gels display the amplification products of immunoglobulins IgH in the top row and IgL k and λ in the middle and bottom rows, respectively. These products were obtained from the second round of nested PCR amplification. Both fresh input material (on the left side of each gel) and frozen samples (on the right side of each gel) show equivalent frequencies and intensities of amplification bands.

Supplementary Figure 4 Minigenes are assembled using polymerase chain reaction (PCR). Polymerase chain reaction (PCR)-generated minigenes are shown. The amplification products corresponding to the assembled minigenes of immunoglobulin heavy chain (IgH), immunoglobulin light chain kappa (IgLκ), and immunoglobulin light chain lambda (IgLλ) are shown in the left, middle, and right panels of the agarose gel electrophoresis images, respectively.
