# Supplementary figures and images for: Rapid generation of human recombinant monoclonal antibodies from antibody-secreting cells using ferrofluid-based technology
Source: Front Immunol. 2024 Apr 18;15:1341389. doi: 10.3389/fimmu.2024.1341389 (PMC11064063; doi:10.3389/fimmu.2024.1341389)

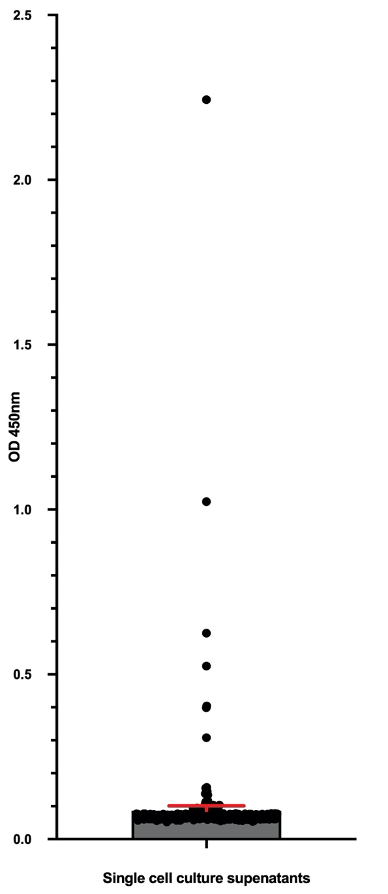

Supplement: Supplementary Figure 1 — Evaluation of IgG secretion in ASC-enriched cultures. The quantitative ELISA graph shows the amount of IgGs produced by a single cell after 16 hours of culture, with each point representing a single result. The assay threshold is set at 200 ng/mL. Human IgG concentrations exceeding 200 ng/mL were detected in more than 4% of the culture supernatants. [file Image_1.tif]

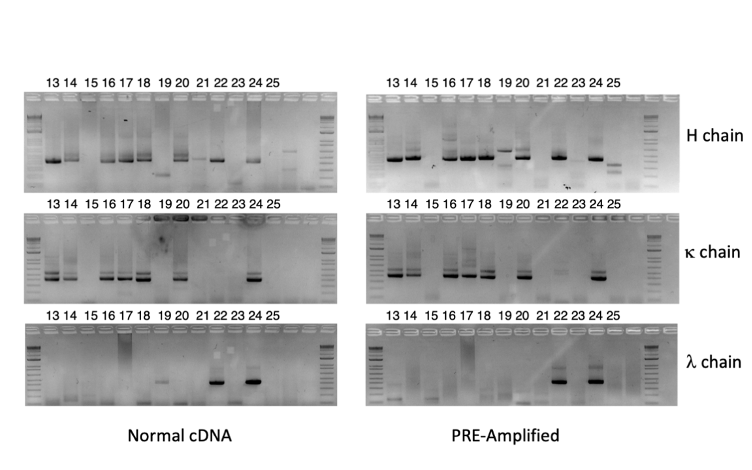

Supplement: Supplementary Figure 2 — Immunoglobulin heavy and light chain variable regions amplified from individual ASC with and without prior cDNA amplification. The amplification products of the immunoglobulin heavy chain (IgH) (top row) and light chains κ and λ (middle and bottom rows, respectively), resulting from the second round of nested PCR, are shown on agarose gels. The left panel of the figure shows the PCR products derived from the cDNA of individual cells, while the right panel shows the amplification resulting from the addition of a pre-amplification step to augment the input material. [file Image_2.tif]

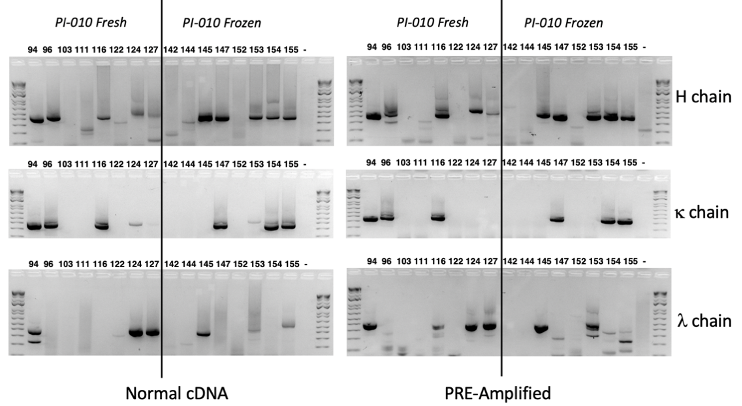

Supplement: Supplementary Figure 3 — Comparison of the recovery of immunoglobulin heavy and light chain variable regions from single antigen-specific ASCs in fresh and frozen PBMCs from the same individual. Agarose gels display the amplification products of immunoglobulins IgH in the top row and IgL k and λ in the middle and bottom rows, respectively. These products were obtained from the second round of nested PCR amplification. Both fresh input material (on the left side of each gel) and frozen samples (on the right side of each gel) show equivalent frequencies and intensities of amplification bands. [file Image_3.tif]

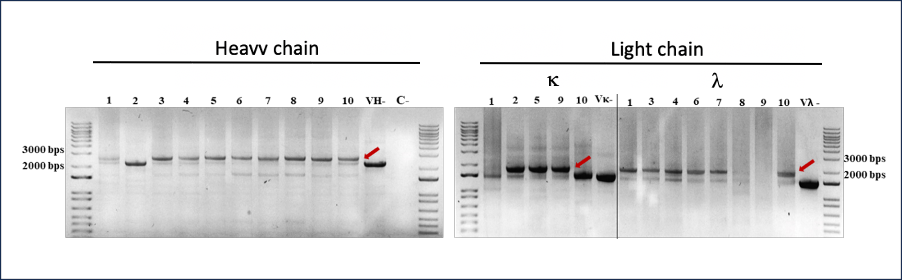

Supplement: Supplementary Figure 4 — Minigenes are assembled using polymerase chain reaction (PCR). Polymerase chain reaction (PCR)-generated minigenes are shown. The amplification products corresponding to the assembled minigenes of immunoglobulin heavy chain (IgH), immunoglobulin light chain kappa (IgLκ), and immunoglobulin light chain lambda (IgLλ) are shown in the left, middle, and right panels of the agarose gel electrophoresis images, respectively. [file Image_4.tif]
